# Supplementary material for: Ultracompact 3D microfluidics for time-resolved structural biology
Source: Nat Commun. 2020 Jan 31;11:657. doi: 10.1038/s41467-020-14434-6 (PMC6994545; doi:10.1038/s41467-020-14434-6)
Supplement: Supplementary file 1 — Supplementary Information [file 41467_2020_14434_MOESM1_ESM.pdf]

Ultracompact 3D microfluidics for structural biology

Juraj Knoška et al.

Supplementary Information

Supplementary Notes

Supplementary Note 1 page 3
Supplementary Note 2 page 3

Supplementary Figures

Supplementary Figure 1: 3D nozzle assembly and setup page 4
Supplementary Figure 2. Nozzle design considerations page 5
Supplementary Figure 3: Nozzle tip sketches and cross sections page 6
Supplementary Figure 4: Dual pulse iLIF jet speed characterization setup page 7
Supplementary Figure 5: Jet speed analysis page 7
Supplementary Figure 6: Inline sample filtering page 8
Supplementary Figure 7: Nozzle development page 9
Supplementary Figure 8: Simulation of gas flow-focusing page 9
Supplementary Figure 9: Hemoglobin crystallization page 10
Supplementary Figure 10: Schematic of diffusive mixing in a T-junction page 10
Supplementary Figure 11: CAD model of the helical mixer page 11
Supplementary Figure 12: Microfluidic X-ray tomography setup page 12
Supplementary Figure 13: Mixing quantification page 13
Supplementary Figure 14: Modular assembly page 14
Supplementary Figure 15: Mixing delay times page 14

Supplementary Tables

Supplementary Table 1: Hemoglobin refinement statistics page 15
Supplementary Table 2: Overview of mix-and-inject experiments page 16

Supplementary Note 1:

Our 3D-printed DFFN reliably jets crystal suspension in vacuum and in atmospheric pressure conditions. Jet acceleration in vacuum is limited due to the quick pressure drop resulting from the rapid expansion of the focusing gas in all directions after exiting the orifice (Supplementary Figure 8A). This pressure drop is strongly reduced when operating under atmospheric pressures. Here jet acceleration extends far out of the gas orifice before the focusing gas has dissipated its kinetic energy (Supplementary Figure 8B). Designing a slow gas-focusing nozzle for atmospheric operating conditions is therefore challenging. Comparably low jet speeds of a few meters per second provide a considerable sample consumption advantage over conventional nozzles running typically >10 m/s in vacuum and even faster at atmospheric pressures for low repetition XFEL sources (LCLS 120 Hz, Sacla 60 Hz, SwissFEL , PAL-XFEL 60 Hz) and state of the art synchrotron sources. Even in a slow GDVNs jet of 10 m/s interrupted by ~50 µm jet explosion gaps[1] more than 99% of the sample is passing between pulses unutilized.

Supplementary Note 2:

The dimensionless Reynolds number of a liquid flow is defined as the ratio of inertial to viscous forces, $Re = \rho v d/\eta$, with liquid density ρ, mean velocity $v = 4Q_{\mathrm{liq}}/\pi d^2$, channel diameter d, and viscosity η. The hydraulic diameter is used in case of non-circular channels defined as $D_{\mathrm{h}} = 4A/P$, with channel cross-sectional area A and wetted perimeter P. In our case of microcrystals suspended in liquid it is necessary to consider the Reynolds number of a particle, $Re_{\mathrm{p}} = Re\, a^2/D_{\mathrm{h}}^2$, with particle diameter a. Inertial effects of particles start to be observable for Re_{p} on the order of 1 or larger.

Our mixer with an inner diameter of 200 µm reaches an open-tube Reynolds number of 8.5 for the largest water flow rate of 80 µl/min. The corresponding particle Reynolds number is 5.3×10^{-3} for a particle diameter of 5 µm.

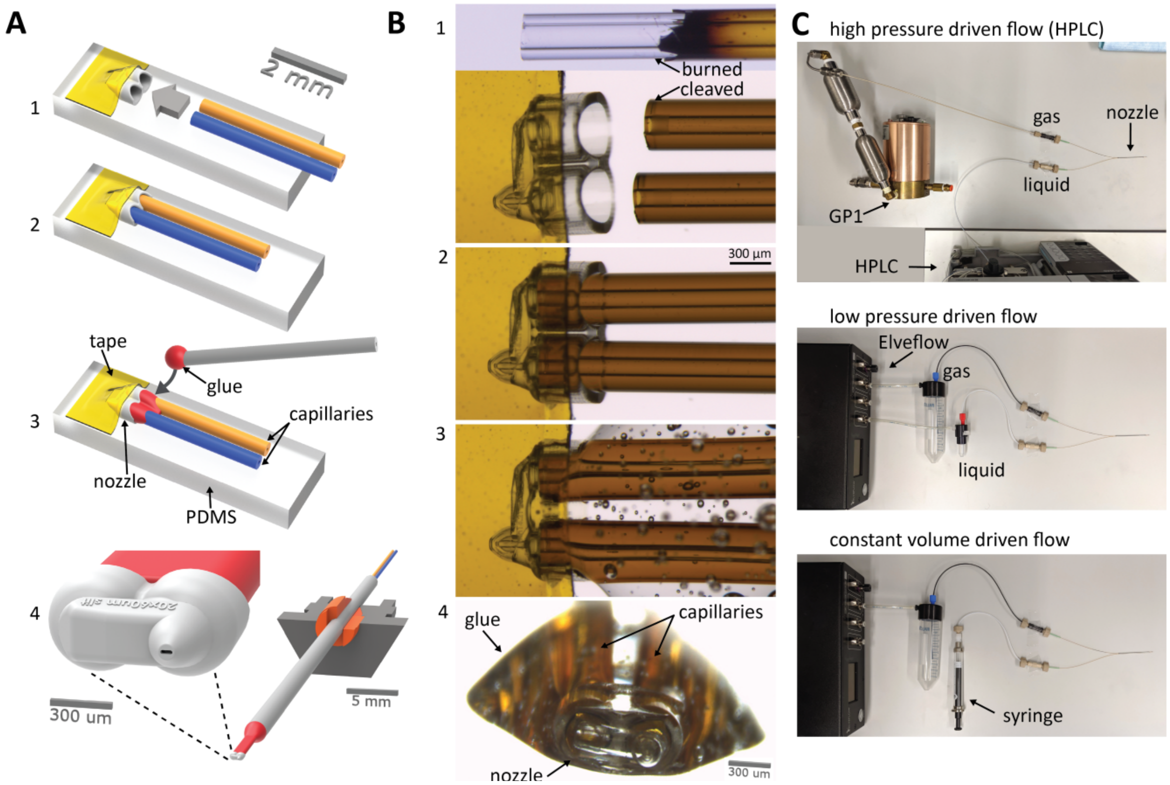

Supplementary Figure 1*. 3D nozzle assembly and setup. Schematic (**A**) and Stereomicrographs (**B**) of nozzle assembly steps. Step 1: After development, fix a dry nozzle on a flat PDMS cushion with a silicon adhesive tape. Step2: Insert glass capillaries into the nozzle ports. To cut a capillary, make a slight incision into the capillary with a ceramic column cutter (60201-318, Thermo Fisher Scientific) using light force. Then, pull the capillary from both sides of the cut until it breaks. A flat end is usually achieved when the capillary breaks with an audible snap. For improved bonding, burn the first few millimeters of the polyimide foil layer around the capillary away with a torch. Step3: Mix five-minute Epoxy (G14250, Thorlabs) for one minute, pre-cure for 70 seconds (±20 seconds, depending on temperature, port and capillary sizes) and then apply a drop of glue on top of the capillaries and the 3D printed nozzle to seal both together. Drag the glue down on the PDMS cushion from both sides of the capillary. Allow the glue to set for at least 10 minutes before displacing the nozzle and gluing a next one. Step 4: Insert and glue this fixed nozzle into a 5 cm long 1/16 inch diameter stainless steel tube (U-138, IDEX) for facile mounting to the SFX nozzle rod. The whole assembly process takes on average 20 minutes per nozzle. (**C**) Photos of simplified setups for liquid jet sample injection. High pressure setup (top) with HPLC pump (LC-20AD, Shimadzu) and gas regulator (GP1, Proportion-air), which is a standard configuration for SFX with a nozzle rod due to the significant pressure drop over the long nozzle capillaries. Typically pressures around 50 bar are required in this configuration. However, low pressure microfluidic systems can be utilized in laboratory experiments as there is no need for long lines. We either use a stand-alone pressure driven pump (Elveflow OB1 MK3 8000, ELVESYS S.A.S) (middle) to independently control the flow of both the liquid (housed in an Eppendorf test tube) and gas (empty 50 ml Falcon tube), or a combination of the pressure module with a syringe pump (Nemesys 290N, Cetoni) to provide constant liquid volume flow for different fluid viscosities (bottom).*

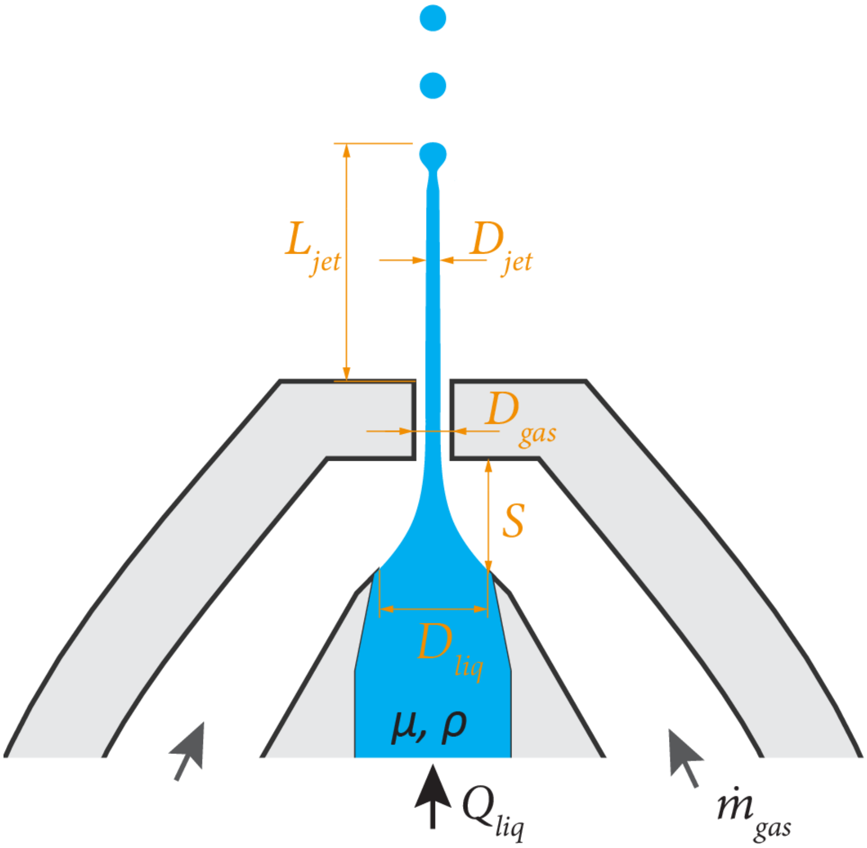

Supplementary Figure 2. *Nozzle design considerations. A schematic of geometrical aspects of the nozzle design with indicated gas orifice diameter,* D_{gas} *, liquid orifice diameter,* D_{liq} *, and spacing between the orifices,* S*. Jetting is primarily characterized by jet length,* L_{jet}*, jet diameter,* D_{jet}*, and it is driven by set liquid volumetric flow rate,* Q_{liq}*, and gas mass flow rate,* $\dot{m}_{\text{gas}}$*. Vega et al.*[2] *empirically related smaller orifice diameters to yield lower minimum flow rates* $Q_{\min} = 2.5 D_{\text{gas}}^{3/4} D_{\text{liq}}^{1/3}\, \rho/\mu$ *, with density,* ρ*, and viscosity,* μ*.* Jet formation and stability strongly depend on nozzle *geometry and properties of the liquid used, in particular viscosity,* μ*, surface tension,* σ*, and density,* ρ*. For instance, surface tension limits liquid motion and has to be overcome by the focusing gas to drive jet formation. The global jetting stability limit is satisfied for the dimensionless Weber number*[3] $We = \frac{8\rho Q^2}{\pi^2 D_{\text{jet}}^3 \sigma} > 1$*. Montanero et al.*[4] *estimated two characteristic minimal flow rates* Q_D *and* Q_v *for the low and high viscosity jetting regimes through a scaling analysis, at which global instability occurs, to be of order* $Q_{\text{D}} = \frac{D_{\text{gas}}\mu}{\rho}$ *, and* $Q_{\text{V}} = \frac{D_{\text{liq}}^2 \sigma}{4\mu}$*. Jetting in the low viscosity regime is limited by recirculation cell mediated viscous friction in the liquid meniscus, hence increasing viscosity liquids require proportionally higher flow rates to jet. In the high viscosity regime, recirculation cells vanish, and increasing viscosity further stabilizes the meniscus and thus the jet.*

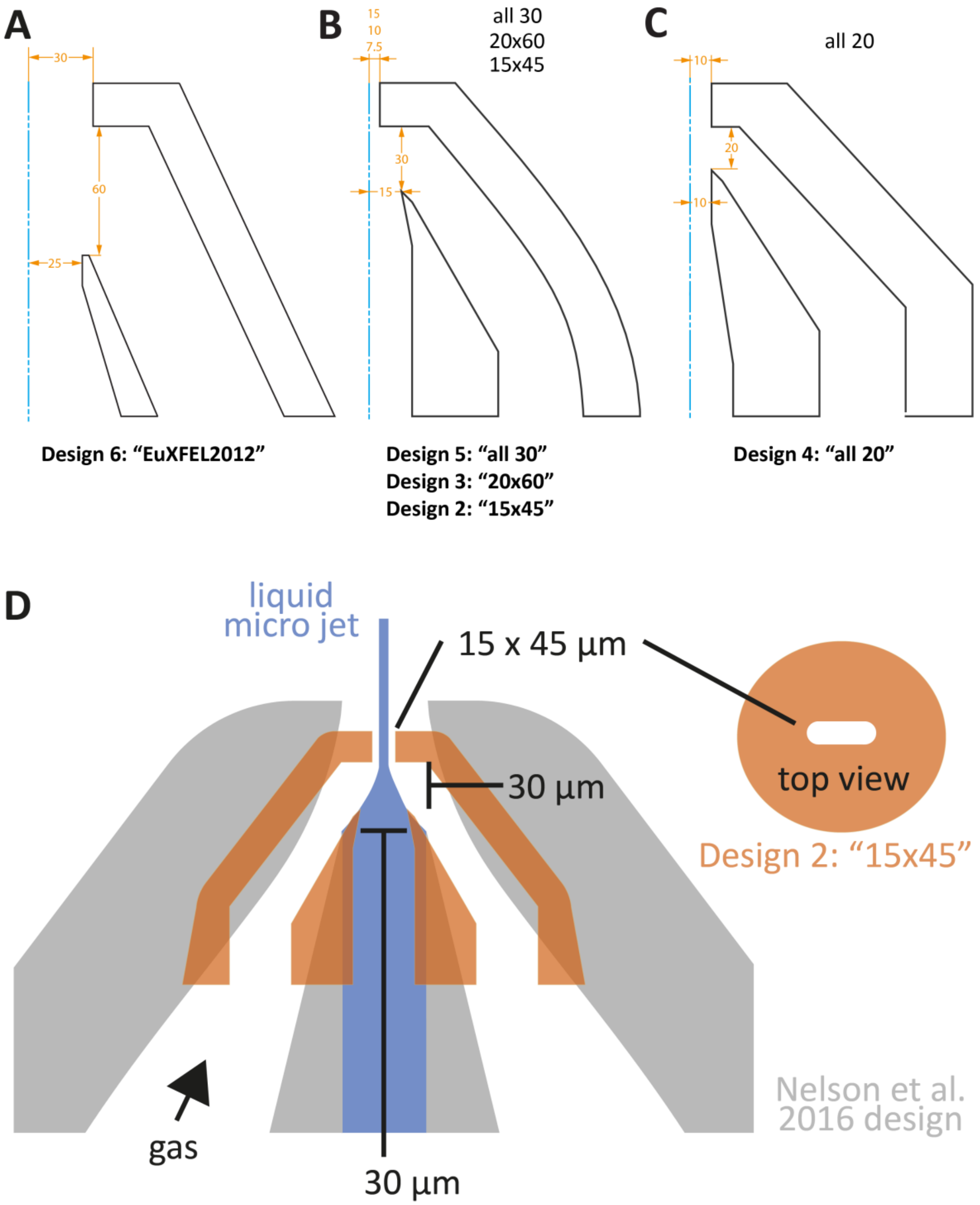

Supplementary Figure 3. *Nozzle tip sketches cross sections. (**A**) Sketch of the Design 6 "EuXFEL2012" nozzle tip with the circular gas orifice. (**B**) Sketches of the nozzle tips for three nozzle designs: nozzle Design 5 with the circular orifice, nozzle Design 3 with the medium sized slit orifice, and Design 2 with the smallest slit orifice. (**C**) Sketch of the nozzle tip of Design 4 with the circular orifice. (**D**) Size-comparison of our 3D printed nozzle tip Design 2 (orange) with the first 3D printed GDVN tip design (gray) that was based on a traditional glass GDVN[5].*

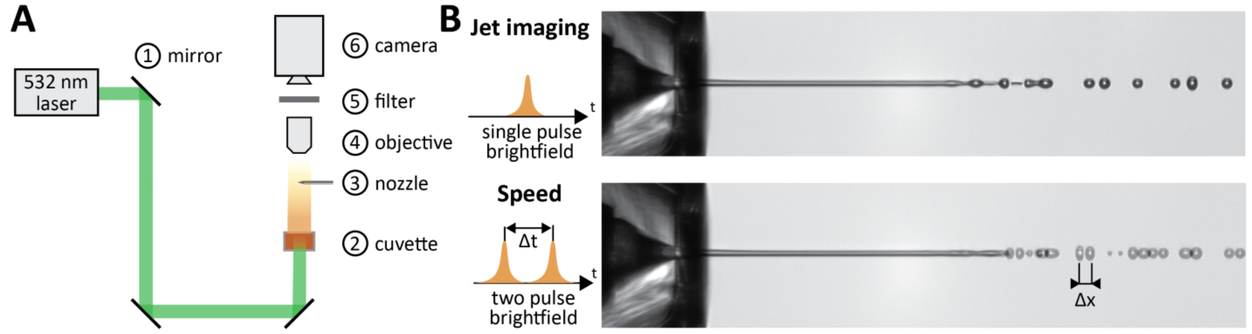

***Supplementary Figure 4**.* Dual pulse iLIF jet speed characterization setup*: (**A**) Schematic of the jet characterization setup used for jet speed measurements. For vacuum PIV, nozzles were mounted in a small vacuum chamber with glass windows as previously described[6]. (**B**) Single pulse iLIF illumination generated in the cuvette in a brightfield configuration is used to acquire high quality speckle-free shadowgraphs of static liquid jets and droplet streams. In vacuum jet speed determination is carried out with dual pulse iLIF illumination with a known delay between the two light flashes by measuring displacement of a droplet. The droplet speed equals to the jet speed in vacuum as there is no additional acceleration present outside of the nozzle tip. However, that's not valid in case of a jet in the atmosphere as the focusing gas continues accelerating liquid jets far past the gas orifice. Therefore, we employ atmospheric PIV using dual pulse laser imaging to trace particles in the jet itself and measure their displacement as a function of distance from the orifice.*

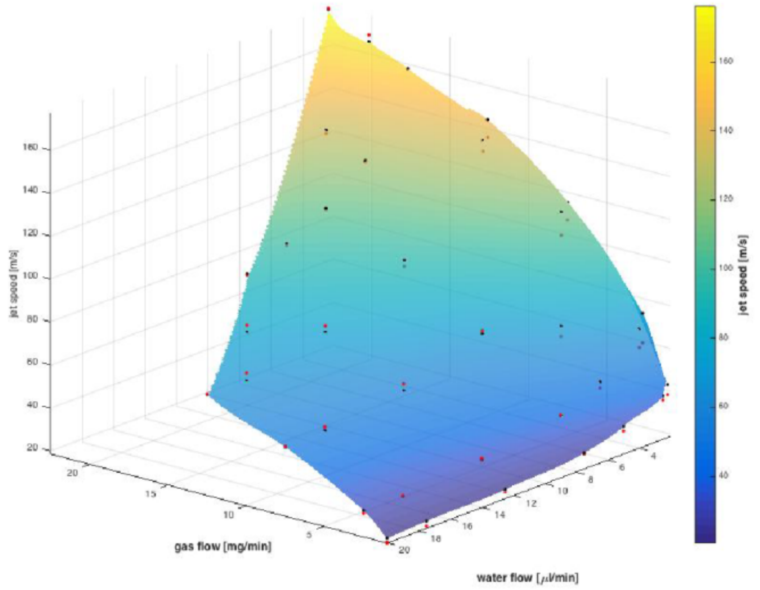

Supplementary Figure ***5****. Jet speed analysis. All measured jet speeds for Design 2 can be fitted into the relation* $v_{\text{jet}} \sim \alpha\, \dot{m}_{\text{gas}}^{1/2} Q_{\text{liq}}^{-1/3}$ *with alpha ~50. Deviation between the corresponding fit (red dots) of the respective isosurface to the measured data (black dots) is minor.*

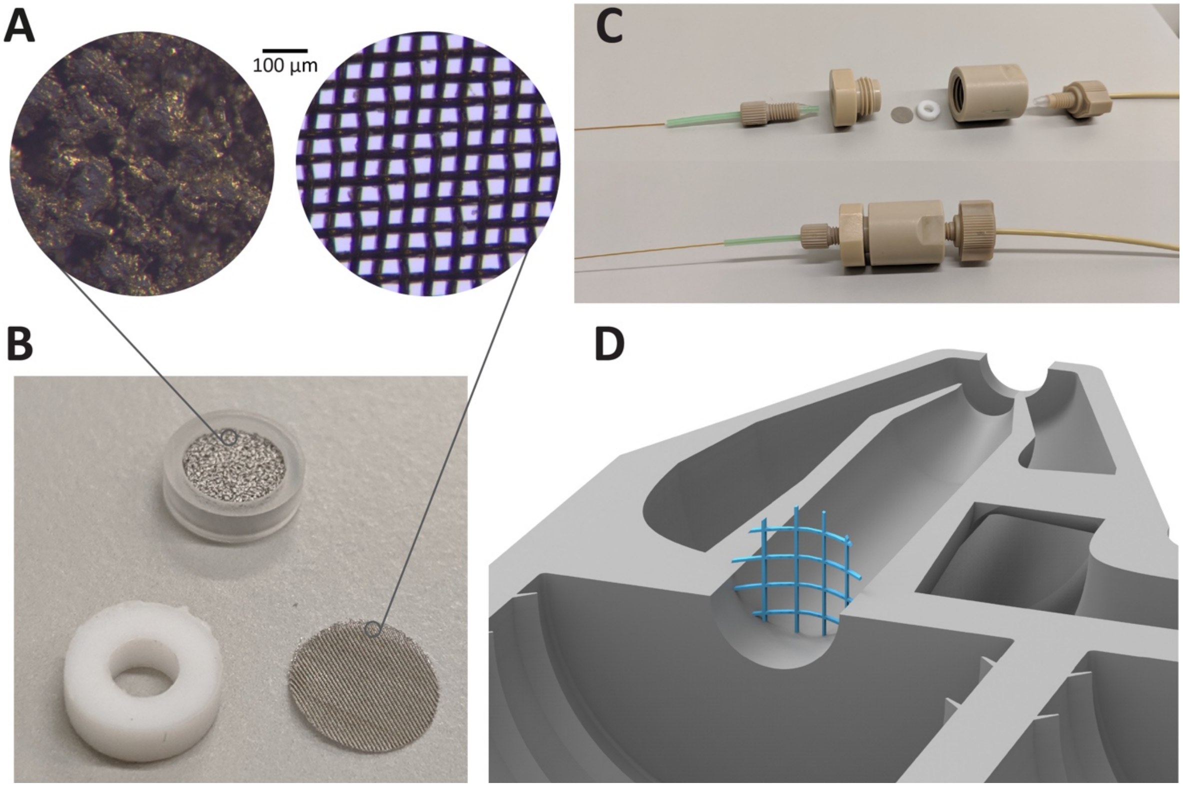

Supplementary Figure 6. *Inline sample filtering. (**A**) Micrograph of commercial 20 µm frit filter (left) and our custom 33 µm stainless steel mesh filter (right). The mesh improves crystal yield after filtering, especially for physically delicate crystals, due to the simplified and reduced shear flow path. (**B**) Comparison of the frit filter (top) (A-122, IDEX) to the mesh filter with Teflon washer (bottom). The mesh (400X400T0012, TWP Inc.) is cut out into a disk shape using a 6-mm hole punch. (**C**) The filter assembly for sample prefiltering or inline filtering contains the mesh filter inside a PEEK body (A-355, IDEX) and two fittings (F-333N or F-300, IDEX). In SFX beamtimes, a prefiltered sample is usually injected and inline filters are only used when clogging is too frequent or when nozzles with small orifices are used. Clogged nozzles were replaced by un-used devices. (**D**) 3D printed inline filters (blue) (Design 6) can be integrated directly into nozzle design (gray) to further safeguard against orifice clogging and therefore extend the operational time of nozzles, particularly those with small orifices.*

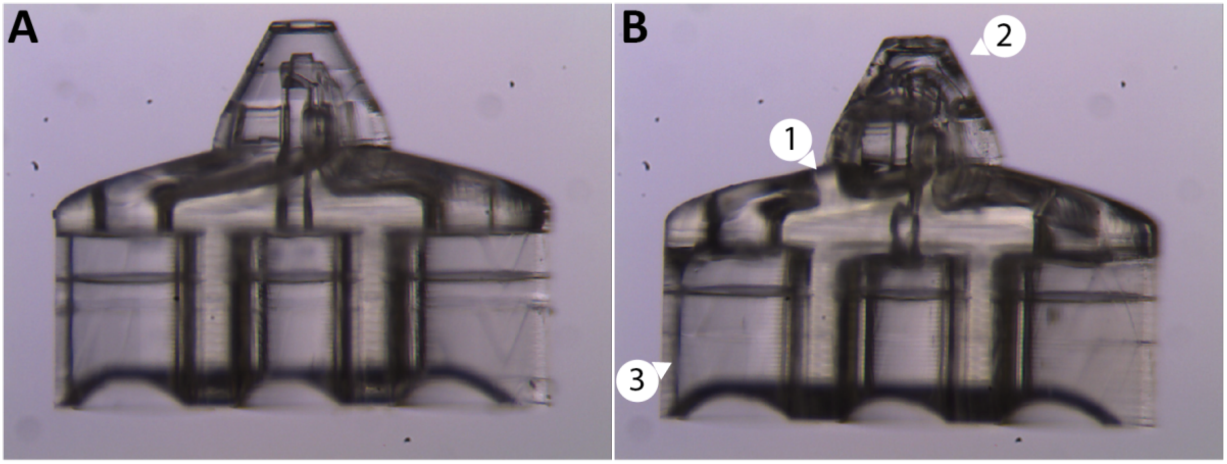

Supplementary Figure 7. *Nozzle development. (**A**) Properly developed nozzle after one day of development, printed using standard recipe. All the channels are clear. (**B**) Underdeveloped nozzle after one day of development, printed with excessive laser power. Channels are clogged with residual resin (1), larger cavities often develop web-like features (2), and only the open areas are well cleared (3).*

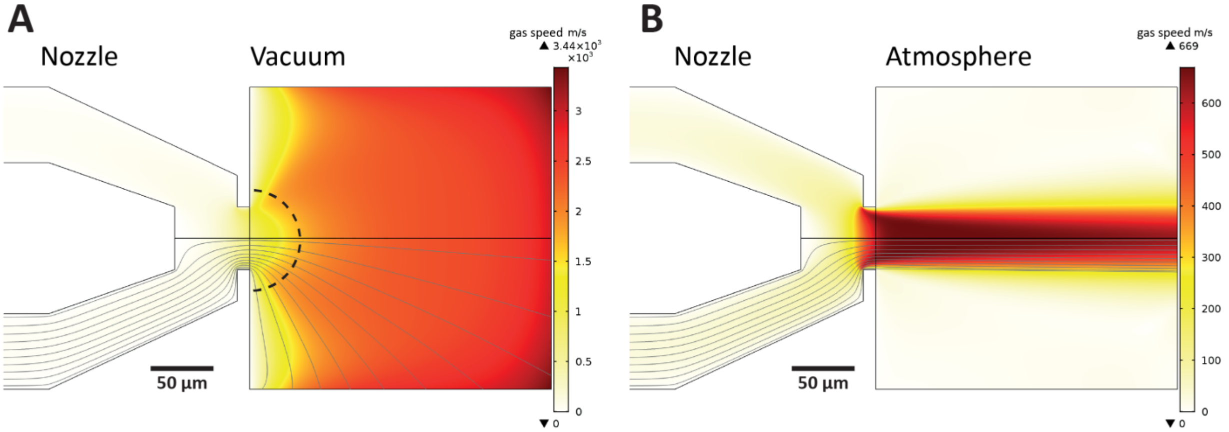

Supplementary Figure 8. *Simulation of gas flow-focusing for vacuum and atmospheric pressure conditions. (**A**). The gas quickly diverges out of the nozzle in a vacuum environment, limiting the acceleration of a liquid jet to a close proximity of the gas orifice. The gas streamlines are represented by the gray lines and the gas speed is color-coded. (**B**) On the other hand, the gas flows out of the gas orifice in a collimated fashion for a nozzle running in atmospheric pressure, thus it continues accelerating a liquid jet even far away from the nozzle. Note that the vacuum case has five times greater maximum speed than the atmospheric case. To simulate qualitative differences of the characteristic gas flow fields emitting from a GDVN into air or vacuum environments, a 2D axisymmetric model of a nozzle and laminar flow regime with a compressible flow model in COMSOL Multiphysics® was used. As laminar flow is no longer valid under vacuum conditions, only a simulation of the gas flow close to the gas orifice was possible, where the focusing gas retains residual pressure. The model might be nonphysical after the dashed line.*

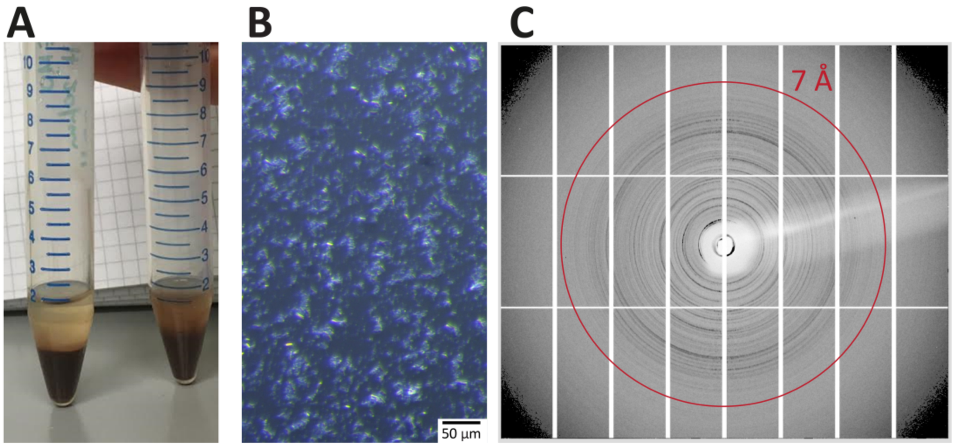

Supplementary Figure 9. *Hemoglobin crystallization. (**A**) Picture of the hemoglobin sample after crystallization where the crystals have settled on the bottom of the falcon tubes. (**B**) Micrograph of the crystalline slurry with crystal sizes down to one micrometer. (**C**) The diffraction quality of the microcrystals was checked at a synchrotron by powder diffraction of pelleted microcrystals. The observed diffraction signal extends up to 7 Å which indicates high crystal quality.*

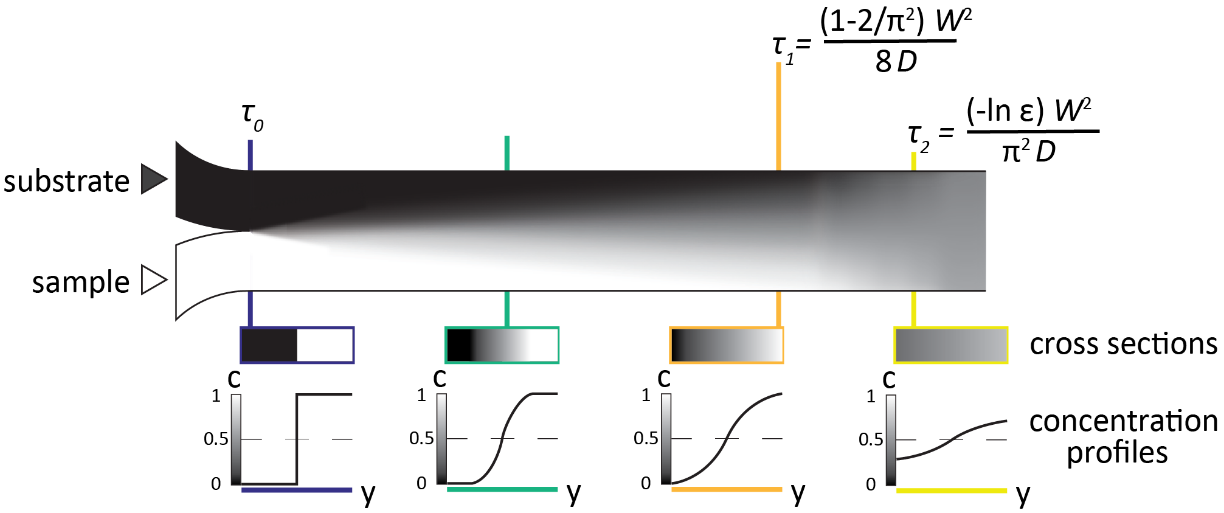

Supplementary Figure 10*. Schematic of diffusive mixing in a T-junction: Diffusion and mixing in microfluidic channels can be computed following an analytical solution for mixing in a T-junction*[7] *as* $U\frac{\partial c}{\partial x} = D\frac{\partial^2 c}{\partial y^2}$ *and* $\frac{\partial c}{\partial \tau} = D\frac{\partial^2 c}{\partial y^2}$ *, assuming steady state concentration* c*, uniform liquid speed* U *across the channel, and neglecting diffusion along the channel, with diffusion coefficient* D*, and directions* x *along and* y *across the channel. In Lagrangian coordinates, with* y *and* $\tau = x/U$ *, this relation can represent the space and time following the fluid resulting in mixing times of* $\tau_1 = \frac{(1-2/\pi^2)W^2}{8D}$ *and* $\tau_2 = \frac{(-\ln\varepsilon)W^2}{\pi^2 D}$ *, with channel width* W*, and the mixing uniformity requirement* $\varepsilon = 2|c - 1/2|_{\max}$*. In this analysis total mixing time corresponds to the sum of* τ_1 *and* τ_2*. The first stage (*τ_1*) of the diffusion process completes when the concentration front reaches the channel wall. The second stage (*τ_2*) downstream, is when the maximum concentration* c *decreases. For ideal, uniform mixing,* $\varepsilon = 0$*. However, depending on ligand solubility and its binding equilibrium with the enzyme, it is feasible to saturate near all binding sites inside the microcrystal when the surrounding bulk fluid has reached about 20% (*$\varepsilon = 0.6$*) of admixed ligand concentration for reaction initiation*[8]*. Using experimental values from Stagno et al.*[9], where $W = 75\ \mu m$, $\varepsilon = 0.6$, and $D = 3.5 \times 10^{-10}\ m^2 s^{-1}$*, we calculate* $\tau_1 = 0.27\ s$ *,* $\tau_2 = 0.83\ s$ *giving the total mixing time of 1.1 s.*

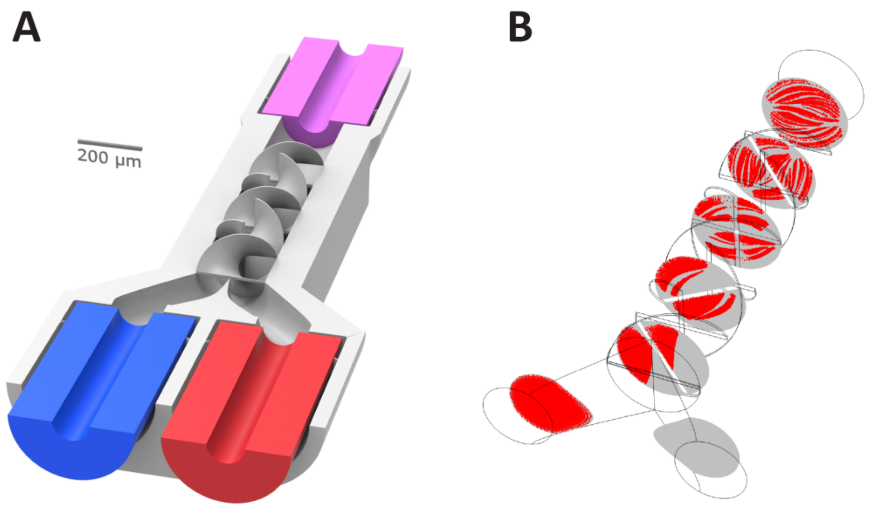

Supplementary Figure 11. *(**A**) CAD model of the helical mixer for actual utilization during TR-SFX experiments (Design 10). Inlet capillaries are in blue and red, while the outlet capillary is depicted as pink. (**B**) Mixing dynamics inside the 3D mixer were modelled using COMSOL Multiphysics® (Fig 5). Modeling diffusion based on numerical simulation using transport of diluted species in a 3D structure with no symmetries becomes computationally demanding technically due to the extremely small mesh size required for diffusion to become mesh independent and hence physically accurate. If a coarser mesh is used, a non-physical diffusion of error is mostly observed. Mixer characterization using particle tracking is much less demanding computationally, as the liquid flow can be accurately resolved using larger mesh elements. In our simulations we use a single phase laminar flow model to calculate steady state liquid flow through the static mixer, and time-resolved tracking of massless particles to estimate mixing dynamics through flow-refolding action.*

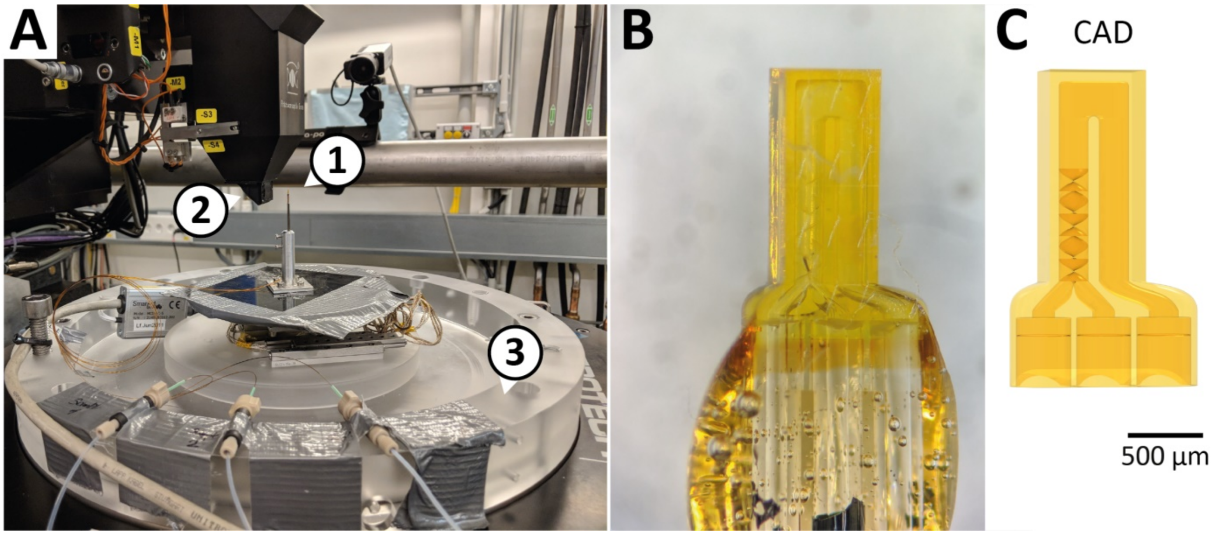

Supplementary Figure 12. *Microfluidic X-ray tomography setup. (**A**) Photograph of the tomography setup at the P05 beamline at PETRAIII with the sample holder configured for tomographic mixing analysis. (1) Microfluidic mixer mounted on the sample holder. (2) Scintillator screen converting X-rays into visible light with a set of magnification optics above it. (3) Rotational stages covered in the waterproof plastic cap. (**B**) Micrograph of the 3D printed mixer after tomographic data collection. Exposed regions of the 3D-printed mixer stained yellow due to X-ray exposure, however no structural defects were observed. (**C**) CAD drawing of the mixer (Design 11) in the respective orientation. There are three parallel ports for connecting capillaries from the bottom with two inlets on the left and one outlet on the right.*

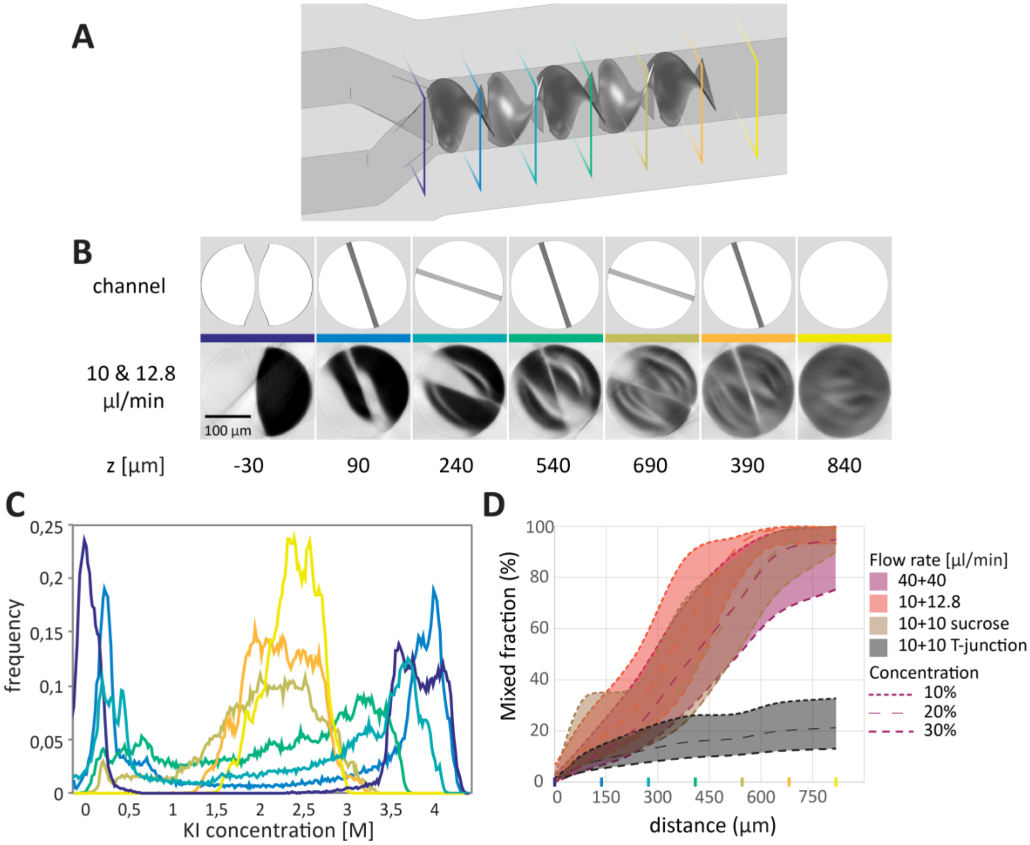

Supplementary Figure 13. *Mixing quantification. (**A**) The cross sections of the tomogram are color-coded according to their position in the mixer (Design 10-12). (**B**) We quantified a cross section upstream of the mixer, for every one of the five mixing elements, and downstream of the mixing elements. The CAD-design cross section of the channel is shown above the corresponding tomogram sections. (**C**) Mixing was analyzed by first creating a histogram of pixel intensity values for each cross section of the flow channel excluding channel walls. Upstream of the mixer (dark blue), two distinct peaks in the histogram, corresponding to the two initial KI concentrations, are clearly visible. With each subsequent mixing element both peaks converge into ultimately a single peak (yellow). (**D**) The mixed fraction was quantified as the relative cross-sectional area that reached a threshold concentration of 10, 20 and 30 % of the initial 4 M KI concentration for each XY section.*

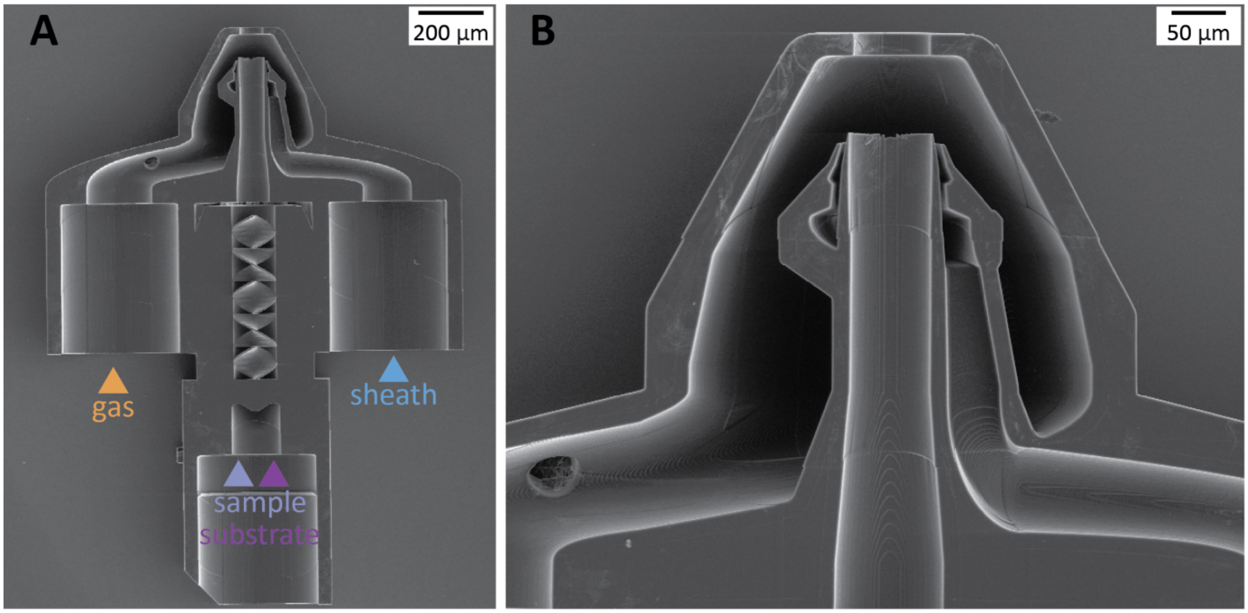

Supplementary Figure 14. *Modular assembly. (**A**) Scanning electron micrograph of a double-flow focused nozzle (Design 8) with an integrated mixer (Design 12). The device is printed halfway through for better visualization of the internal channels. (**B**) Magnified view of the nozzle tip.*

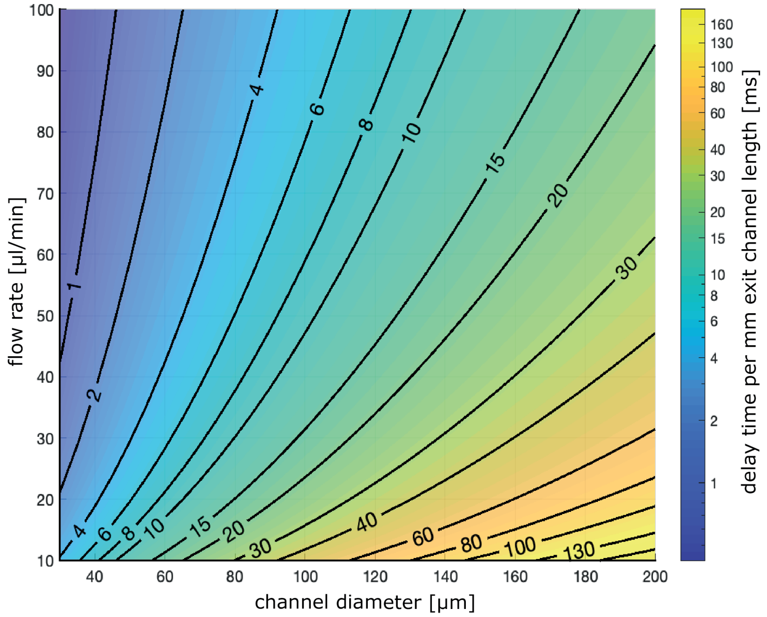

Supplementary Figure 15. *Theoretical mixing delay times plotted as a function of channel diameter and flow rate for a 1 mm exit channel segment. By adjusting total flow rate, channel diameter and/or length a range of delay times from the sub-millisecond range to several seconds can be obtained. This allows tailoring of mixing injectors for investigating reaction intermediates that occur over various different time scales. For instances, the direct insert mixing-DFFN arrangement as depicted in the Supplementary Figure 14 has about 2.13 nL exit channel volume which adds about 1.6 ms delay time at 80 µl/min average flow rate.*

Supplementary Table 1. *Hemoglobin refinement statistics*

Photon energy (mean value)	7150 eV
Pulse length	10 fs
Space group	$P\ 2_1\ 2_1\ 2_1$
Unit cell	
a, b, c	62.5 80.8 110.8 Å
α, β, γ	90, 90, 90 °
# of hits/indexed lattices	140443 / 114958
Number of unique reflections	21072 (2074)
Resolution range	20.0 - 2.46 (1.82 -1.76) Å
Completeness	100 (100) %
Multiplicity	1059 (99.5)
R_{split}	0.058 (0.538)
$I/\sigma(I)$	15.11 (2.06)
$CC_{1/2}$	0.99 (0.70)
Wilson B-factor	40.70 Å^2
R_{Work}	0.166 (0.253)
R_{Free}	0.213 (0.287)
Rmsd bonds/ Rmsd angles	0.005 Å / 0.98 °
Ramachandran favored	98.94 %
Ramachandran allowed	1.06 %
Ramachandran outliers	0.00 %
Clashscore	2.98
Average B-factor	50.5 Å^2
Macromolecules	50.5 Å^2
Ligands	52.7 Å^2
Solvent	45.2 Å^2
Number of TLS groups	15
PDB code	6R2O

***Supplementary Table 2**. Overview of mix-and-inject experiments*

System	Lysozyme + chitotriose	RNA riboswitch + adenine	ß-lactamase + ceftriaxone	ß-lactamase + ceftriaxone	
				Shards crystal form	Needle crystal form
**DOI**	doi:10.1107/S2052 252517013124	doi:10.1038/ nature20599	doi: 10.1063/ 1.4972069	doi:10.1186/s12915-018-0524-5	
**Device**	on tape	T-junction	T-junction	Flow-focusing injector doi: 10.1063/1.4961971	
**Diffusion coef [cm²/s]**	3.6×10^{-6}		2.74×10^{-6} doi: 10.1007/s10008-008-0567-6	2.3×10^{-6} doi: 10.1039/C0AY00582G	
**Mixing ratios**	1:1	1:1	1:4	1:16.5	
**Mixing time [ms]**	2 s (50s point) 80 ms (2s point) (estimated*)	1 s (estimated*)	100 ms (estimated*)	5 ms	
**Min delay time [s]**	2	10	1.4-2.6	0.03	
**Max delay time [s]**	50	600		2	
**Capillary ID [µm]**	100	75	50	75	
**flow rate [µl/min]**	N/A	30	75	70	
**Crystal size**	6-8 µm	5 µm average plates 1-10 µm long		$10 \times 10 \times 3$ µm³ shards	$5 \times 2 \times 2$ µm³ needles
**PDB**	50 s 5njr, 5njs 2 s 5njq, 5njp	APO 5E54, 10 s 5SWD, 600 s 5SWE	N/A	30 ms 6B5Y	30 ms 6B6C
**Solvent Content**	41.90%	45.81% (intermediate) 42.69% (ligand bound)	N/A	65.20%	36.91%

*Estimated following an analytical solution for mixing in a T-junction as detailed in Supplementary Figure 10

Supplementary References

1 C. A. Stan, D. Milathianaki, H. Laksmono, R. G. Sierra, T. A. McQueen, M. Messerschmidt, G. J. Williams, J. E. Koglin, T. J. Lane, M. J. Hayes, S. A. H. Guillet, M. Liang, A. L. Aquila, P. R. Willmott, J. S. Robinson, K. L. Gumerlock, S. Botha, K. Nass, I. Schlichting, R. L. Shoeman, H. A. Stone and S. Boutet, *Nat. Phys.*, 2016, **12**, 966–971.

2 E. J. Vega, J. M. Montanero, M. A. Herrada and A. M. Gañán-Calvo, *Phys. Fluids*, 2010, **22**, 1–10.

3 A. M. Gañán-Calvo and J. M. Montanero, *Phys. Rev. E - Stat. Nonlinear, Soft Matter Phys.*, 2009, **79**, 1–18.

4 J. M. Montanero, N. Rebollo-Muñoz, M. A. Herrada and A. M. Gañán-Calvo, *Phys. Rev. E*, 2011, **83**, 036309.

5 G. Nelson, R. A. R. A. Kirian, U. Weierstall, N. A. N. A. N. A. Zatsepin, T. Faragó, T. Baumbach, F. Wilde, F. B. P. F. B. P. F. B. P. Niesler, B. Zimmer, I. Ishigami, M. Hikita, S. Bajt, S.-R. S. Yeh, D. L. D. L. Rousseau, H. N. Chapman, J. C. H. J. C. H. Spence, M. Heymann, T. Faragó, T. Baumbach, F. Wilde, F. B. P. F. B. P. F. B. P. Niesler, B. Zimmer, I. Ishigami, M. Hikita, S. Bajt, S.-R. S. Yeh, D. L. D. L. Rousseau, H. N. Chapman, J. C. H. J. C. H. Spence and M. Heymann, *Opt. Express*, 2016, **24**, 73–77.

6 K. R. Beyerlein, L. Adriano, M. Heymann, R. A. Kirian, J. Knoska, F. Wilde, H. N. Chapman, S. Bajt, J. Knoška, F. Wilde, H. N. Chapman and S. Bajt, *Rev. Sci. Instrum.*, 2015, **86**, 125104.

7 Y. K. Suh and S. Kang, *Micromachines*, 2010, **1**, 82–111.

8 J. L. Olmos, S. Pandey, J. M. Martin-Garcia, G. Calvey, A. Katz, J. Knoska, C. Kupitz, M. S. Hunter, M. Liang, D. Oberthuer, O. Yefanov, M. Wiedorn, M. Heyman, M. Holl, K. Pande, A. Barty, M. D. Miller, S. Stern, S. Roy-Chowdhury, J. Coe, N. Nagaratnam, J. Zook, J. Verburgt, T. Norwood, I. Poudyal, D. Xu, J. Koglin, M. H. Seaberg, Y. Zhao, S. Bajt, T. Grant, V. Mariani, G. Nelson, G. Subramanian, E. Bae, R. Fromme, R. Fung, P. Schwander, M. Frank, T. A. White, U. Weierstall, N. Zatsepin, J. Spence, P. Fromme, H. N. Chapman, L. Pollack, L. Tremblay, A. Ourmazd, G. N. Phillips and M. Schmidt, *BMC Biol.*, 2018, **16**, 59.

9 J. R. Stagno, Y. Liu, Y. R. Bhandari, C. E. Conrad, S. Panja, M. Swain, L. Fan, G. Nelson, C. Li, D. R. Wendel, T. A. White, J. D. Coe, M. O. Wiedorn, J. Knoska, D. Oberthuer, R. A. Tuckey, P. Yu, M. Dyba, S. G. Tarasov, U. Weierstall, T. D. Grant, C. D. Schwieters, J. Zhang, A. R. Ferré-D'Amaré, P. Fromme, D. E. Draper, M. Liang, M. S. Hunter, S. Boutet, K. Tan, X. Zuo, X. Ji, A. Barty, N. A. Zatsepin, H. N. Chapman, J. C. H. Spence, S. A. Woodson, Y.-X. Wang, J. R. Stagno, Y. Liu, Y. R. Bhandari, C. E. Conrad, S. Panja, M. Swain, L. Fan, G. Nelson, C. Li, D. R. Wendel, T. A. White, J. D. Coe, M. O. Wiedorn, J. Knoska, D. Oberthuer, R. A. Tuckey, P. Yu, M. Dyba, S. G. Tarasov, U. Weierstall, T. D. Grant, C. D. Schwieters, J. Zhang, A. R. Ferré-D'Amaré, P. Fromme, D. E. Draper, M. Liang, M. S. Hunter, S. Boutet, K. Tan, X. Zuo, X. Ji, A. Barty, N. A. Zatsepin, H. N. Chapman, J. C. H. Spence, S. A. Woodson and Y.-X. Wang, *Nature*, 2017, **541**, 242–246.
