# Supplementary material for: Ultracompact 3D microfluidics for time-resolved structural biology
Source: Nat Commun. 2020 Jan 31;11:657. doi: 10.1038/s41467-020-14434-6 (PMC6994545; doi:10.1038/s41467-020-14434-6)
Supplement: Supplementary file 3 — Description of Additional Supplementary Files [file 41467_2020_14434_MOESM3_ESM.pdf]

Description of Additional Supplementary Files

Design file list

File name: Supplementary Data 1
Description: Design 1 (jkmh #1) Mini nozzle for 160 µm OD capillaries with 40 µm gas orifice

File name: Supplementary Data 2
Description: Design 2 (jkmh #2) Slit orifice nozzle, 15x45, 30, 30 (gas orifice, spacing, sample orifice in µm)

File name: Supplementary Data 3
Description: Design 3 (jkmh #3) Slit orifice nozzle, 20x60, 30, 30

File name: Supplementary Data 4
Description: Design 4 (jkmh #4) Circular orifice nozzle, 20, 20, 20

File name: Supplementary Data 5
Description: Design 5 (jkmh #5) Circular orifice nozzle, 30, 30, 30

File name: Supplementary Data 6
Description: Design 6 (jkmh #6) EuXFEL2012 nozzle design, 60, 60, 50

File name: Supplementary Data 7
Description: Design 7 (jkmh #7) Nozzle tip filter, 20 µm mesh

File name: Supplementary Data 8
Description: Design 8 (jkmh #8) Double-flow focusing nozzle 70, 70, 75

File name: Supplementary Data 9
Description: Design 9 (jkmh #9) Double-flow focusing nozzle 70, 55, 50

File name: Supplementary Data 10
Description: Design 10 (jkmh #10) Inline helical mixer for long time-delays with 200 µm ID

File name: Supplementary Data 11
Description: Design 11 (jkmh #11) U-turn helical mixer adapted for tomography with 200 µm ID

File name: Supplementary Data 12
Description: Design 12 (jkmh #12) Helical mixer insert for a nozzle, 200 µm ID

Supplementary Movies

File name: Supplementary Movie 1
Description: 3D printing process. Real time video of the printing process for our miniature nozzle Design 1.

File name: Supplementary Movie 2
Description: Nozzle jetting – stable. Stable jet of liquid water captured using dual-pulse illumination with 500 ns delay between pulses. The nozzle (Design 2) ran with a water flow of 17.5 µl/min and a helium gas flow of 1.1 mg/min in vacuum, resulting in a jet diameter of 4.1 µm and a jet speed of 22 m/s.

File name: Supplementary Movie 3
Description: Nozzle jetting – metastable. Metastable jet of liquid water captured using dual-pulse illumination with 35 ns delay between pulses. The nozzle (Design 2) ran with a water flow of 7.5 µl/min and a helium gas flow of 20 mg/min in vacuum. The double jet is an artefact caused by reflections induced by a low-quality glass window on our vacuum chamber.

File name: Supplementary Movie 4
Description: Nozzle jetting – unstable. Unstable jet of liquid water captured using dual-pulse illumination with 20 ns delay between pulses. The nozzle (Design 2) ran with a water flow of 2.4 µl/min and a helium gas flow of 27.5 mg/min in vacuum resulting in a jet speed of 193 m/s. The double jet is an artefact caused by reflections induced by a low-quality glass window on our vacuum chamber.

File name: Supplementary Movie 5
Description: Nozzle jetting – fast and thin. The thinnest and fastest stable liquid jet of water captured using dual-pulse illumination with 20 ns delay between pulses. The nozzle (Design 2) ran with a water flow of 2.4 µl/min and a helium gas flow of 22.5 mg/min in vacuum resulting in a jet diameter of 0.54 µm and the jet speed of 177 m/s. The double jet is an artefact caused by reflections induced by a low-quality glass window on our vacuum chamber.

File name: Supplementary Movie 6
Description: DFFN crystals in the jet. Hemoglobin crystals (~1 µm) were imaged in the jet produced by DFFN nozzle (Design 8) flowing at 5 µl/min with additional ethanol sheath flow of 11 µl/min and Helium gas flow of 2.2 mg/min. The jet was positioned slightly out of focus for improved visibility of the hemoglobin microcrystals.

File name: Supplementary Movie 7
Description: Tomogram - flow through the helical mixer for 10+12.8 µl/min. Animation of the horizontal slices which move through the tomogram of the helical mixer (Design 10-12) in the direction of the liquid flow. The aqueous solution of potassium iodide (contrast agent - black) was supplied at 12.8 µl/min and mixed with water (white) flowing at 10 µl/min as they travelled through the helical mixing elements that induce splitting and stretching of the liquids. The complete 3D reconstruction with the slice position indicator is included on the right side.

File name: Supplementary Movie 8
Description: Tomogram - flow through the helical mixer for 40+40 µl/min. Animation of the horizontal tomogram sections through the helical mixer (Design 10-12) in the direction of the liquid flow. The aqueous solution of potassium iodide (contrast agent - black) was supplied at 40 µl/min and mixed with water (white) flowing at 40 µl/min as they travelled through the helical mixing elements that induce splitting and stretching of the liquids. The complete 3D reconstruction with the slice position indicator is included on the right side.
